# 外周血检测指标联合TCF1^+^CD8^+^ T淋巴细胞占比预测中晚期肺癌免疫治疗疗效及预后的临床队列研究

**DOI:** 10.3779/j.issn.1009-3419.2023.102.29

**Published:** 2023-08-20

**Authors:** Hong LUO, Sisi DAI, Yalun LI, Panwen TIAN, Qintong LI, Xuyu CAI

**Affiliations:** ^1^610041 成都，四川大学华西第二医院妇产科、检验科、儿科，出生缺陷与妇幼相关疾病教育部重点实验室，发育与妇幼相关疾病四川省重点实验室，生长、代谢与衰老研究中心，生物治疗国家重点实验室和生物治疗协同创新中心，疾病分子网络前沿科学中心，四川大学华西医院; ^1^Departments of Obstetrics & Gynecology, Laboratory Medicine and Pediatrics, West China Second University Hospital; Key Laboratory of Birth Defects and Related Diseases of Women and Children, Ministry of Education, Development and Related Diseases of Women and Children Key Laboratory of Sichuan Province, Center of Growth, Metabolism and Aging, State Key Laboratory of Biotherapy and Collaborative Innovation Center of Biotherapy, Frontiers Science Center for Disease-related Molecular Network, West China Hospital, Sichuan University; ^2^四川大学华西医院呼吸健康研究所，四川大学疾病分子网络前沿科学中心，四川大学华西医院; ^2^Institute of Respiratory Health, Frontiers Science Center for Disease-related Molecular Network, West China Hospital, Sichuan University; ^3^四川大学华西医院老年医学中心; ^3^Center of Gerontology and Geriatrics, West China Hospital, Sichuan University; ^4^四川大学华西医院呼吸与危重症医学科，呼吸健康与多发病国家重点实验室，四川省精准医学重点实验室; ^4^Department of Pulmonary and Critical Care Medicine, State Key Laboratory of Respiratory Health and Multimorbidity, Precision Medicine Key Laboratory of Sichuan Province, West China Hospital, Sichuan University; ^5^四川大学华西医院肺癌中心; ^5^Lung Cancer Center, West China Hospital,Sichuan University, Chengdu 610041, China

**Keywords:** 肺肿瘤, 免疫检查点抑制剂, 外周血, 疗效, 预后, Lung neoplasms, Immune checkpoint inhibitors, Peripheral blood, Efficacy, Prognosis

## Abstract

**背景与目的:**

免疫检查点抑制剂治疗目前缺乏可用于预测疗效的生物标志物，本研究旨在探讨外周血实验室检查指标联合淋巴细胞亚群分布与中晚期肺癌免疫治疗疗效及预后的相关性。

**方法:**

前瞻性纳入2021年5月至2023年7月四川大学华西医院接受一线免疫治疗的中晚期肺癌患者，收集患者临床信息，同时采用流式细胞术检测患者治疗前后外周血淋巴细胞亚群。分别采用Logistic回归分析和Cox模型探究疗效和预后的影响因素。

**结果:**

Logistic回归表明基线转录因子T细胞因子1（transcription factor T cell factor 1, TCF1）^+^CD8^+^ T细胞占比及治疗1个周期后外周血白细胞计数、淋巴细胞百分比、细胞角蛋白19片段（cytokeratin 19 fragment, CYFRA21-1）水平、中性粒细胞淋巴细胞比率（neutrophil-lymphocyte ratio, NLR）是疗效的影响因素（P<0.05）。Cox回归分析显示基线TCF1^+^CD8^+^ T细胞占比（P=0.020）、治疗1个周期后外周血白细胞计数（P<0.001）是预后的影响因素。

**结论:**

基线高水平TCF1^+^CD8^+^ T细胞占比和治疗1个周期后低水平白细胞计数和CYFRA21-1可能是患者从免疫治疗中获益的有利因素。

## Abstract (en)

**Background and objective:**

Immune checkpoint inhibitors (ICIs) therapy lacks viable biomarkers for response and prognosis prediction. This study aimed to investigate the correlation of peripheral blood laboratory test results combined with lymphocyte subset ratios to the response and prognosis of immunotherapy in advanced lung cancer.

**Methods:**

Advanced lung cancer patients admitted to West China Hospital, Sichuan University from May 2021 to July 2023 were prospectively enrolled in this study. Clinical data and peripheral blood were collected before and after treatment and lymphocyte subset ratios were analyzed by flow cytometry. Logistic regression was used to identify factors correlated to ICIs treatment efficacy. Cox modeling was applied to explore the prognostic factors.

**Results:**

Logistic regression showed that the baseline level of transcription factor T cell factor 1 (TCF1)^+^CD8^+^ T cell ratio and peripheral white blood cell (WBC) count, lymphocyte percentage, cytokeratin 19 fragment (CYFRA21-1) after 1 cycle of ICIs treatment were the potential predictors for ICIs response (P<0.05). Cox regression analysis showed that the baseline level of TCF1^+^CD8^+^ T cell ratio (P=0.020) and peripheral WBC count after 1 cycle of ICIs treatment (P<0.001) were prognostic factors.

**Conclusion:**

Patients with high baseline TCF1^+^CD8^+^ T cell ratio combined with low WBC counts and low CYFRA21-1 level after 1 cycle of ICIs treatment are more likely to benefit from ICIs therapy.

肺癌是当今全球最常见的癌症，其发病率和癌症致死率都位居前列^[1]^。多数肺癌患者就诊时已处于疾病晚期，预后较差；以免疫检查点抑制剂（immune checkpoint inhibitors, ICIs）为主的免疫治疗的应用是肿瘤治疗中的一项重大转折，显著提高了晚期非小细胞肺癌患者的临床预后，已成为晚期肺癌患者的一线治疗。但仅有20%肺癌患者从免疫治疗中获益^[2]^，在目前的临床实践中，很难准确筛选患者群体在免疫疗法中持续受益。靶向程序性死亡受体1（programmed death receptor 1, PD-1）和程序性死亡配体1（programmed death ligand 1, PD-L1）是ICIs治疗的主要策略，因此，PD-L1的表达最初被认为是预测抗PD-1/PD-L1治疗反应的合理生物标志物，但现有研究^[3,4]^指出，PD-L1表达无法准确预测免疫治疗疗效。并且，由于PD-L1检测依赖于肿瘤组织，取材限制导致其表达的时空异质性^[5,6]^，更加影响了PD-L1检测的稳定性和准确性^[7]^。另外，组织样本无法多次取样，难以实时检测免疫治疗过程中的变化。因此迫切地需要取材方便的检测方法，以监测免疫治疗全过程的疗效。外周血检测作为临床常规的检查方案，具有简便且创伤小的优势，且能在不同时间点多点取样以达到纵向对比分析并全程监测的目的，其作为探究免疫治疗生物标志物的重要价值正在被不断开发^[8⇓-10]^。除此之外，外周血中免疫细胞状态与接受免疫治疗的肺癌患者预后的关系也被多次报道^[11⇓-13]^。本研究对接受ICIs治疗的晚期肺癌患者外周血实验室检测指标及免疫细胞亚群进行探究，分析其与ICIs治疗疗效及预后的相关性，以期为晚期肺癌免疫治疗疗效预测和预后评估提供新的见解。

## 1 资料与方法

### 1.1 研究设计

本研究前瞻性纳入2021年5月至2023年7月在四川大学华西医院肺癌中心就诊并接受抗PD-1/PD-L1治疗的III-IV期肺癌患者，收集临床病理资料及外周血实验室检查结果。纳入标准：①组织病理学检查确诊为肺癌；②临床分期为III-IV期的肺癌；③基因检测无表皮生长因子受体（epidermal growth factor receptor, EGFR）/间变性淋巴瘤激酶（anaplastic lymphoma kinase, ALK）/c-ros肉瘤致癌因子-受体酪氨酸激酶（ROS proto-oncogene 1, receptor tyrosine kinase, ROS1）等驱动基因突变；④东部合作肿瘤小组（Eastern Cooperative Oncology Group, ECOG）评分≤2分；⑤接受免疫联合化疗作为系统抗肿瘤治疗；⑥未患有需要全身治疗的活动性自身免疫性疾病；⑦未接受其他已批准的全身性免疫调节剂、长期大量使用激素或使用其他免疫抑制剂；⑧临床资料完整且具备至少1年的随访信息。

### 1.2 资料收集

对于所有符合纳入标准的患者，收集临床病理学资料：诊断、性别、年龄、ECOG、吸烟史、肿瘤家族史、病理类型和临床分期和治疗方案。收集患者外周血实验室检查指标包括：外周血白细胞（white blood cell, WBC）计数、外周血淋巴细胞百分比（lymphocyte percentage, LYMPH%）、中性粒细胞淋巴细胞百分比（neutrophil-to-lymphocyte ratio, NLR）及细胞角蛋白19片段（cytokeratin 19 fragment, CYFRA21-1）等。免疫治疗反应根据实体瘤免疫疗效评估标准（modified Response Evaluation Criteria in Solid Tumors 1.1 for immune-based therapeutics, iRECIST 1.1）每周期（3-4周）进行评估，6个月缓解状态依据患者从首次用药开始随访至6个月的全程综合评估，反应者（responders, R）包括完全缓解（complete response, CR）、部分缓解（partial response, PR）和疾病稳定（stable disease, SD）持续时间≥6个月，无反应者（non-responders, NR）定义为在首次用药开始6个月内发生疾病进展（progressive disease, PD），无论最佳疗效如何。预后的评估指标采用无进展生存期（progression-free survival, PFS）。PFS是指从首次接受免疫治疗开始直至影像学或PD或因任何原因死亡的时间。末次随访时间为2023年7月9日，随访时长为369-844 d。

### 1.3 样本采集

对于流式分析队列的所有患者，自愿参与研究并签署书面知情同意书，采集首次用药前3天内的外周血及用药1个周期后的外周静脉血5.0 mL于含有EDTA抗凝剂的真空采血管中，颠倒混匀，转移到离心管中，离心分层后，分离血浆及外周血。将外周血缓慢加入Ficoll液中，4 ^o^C、800 g 20 min，升速=1，降速=1。吸取中间白膜层于5 mL RPMI中，4 ^o^C、500 g、7 min。加入1-2 mL裂红液，室温裂解约3 min后，4 ^o^C、500 g、5 min。PBS清洗，4 ^o^C、500 g、5 min。将细胞重悬于PBS至浓度为2×10^6^/mL，备用。

### 1.4 多色流式细胞术分析

荧光抗体CD3、CD19、CD8、转录因子T细胞因子1（transcription factor T cell factor 1, TCF1）购自美国BD公司；CD4、PD-1购自Biolegend公司；LIVE/DEAD Fixable Blue Dead Cell Stain（L/D）、BV stain buffer购自Sigma-Aldrich公司。355、405、640 nm激光器配置Symphony A5流式细胞仪（美国BD Bioscience）。根据已滴定好的抗体质量浓度及建立的流式检测方案，检测27例晚期肺癌患者接受ICIs治疗前后两次外周血标本。预先将CD3、CD19、CD4、CD8、TCF-1、PD-1抗体和L/D染料混合于50.0 μL BV stain buffer中，抗体加入量按照已滴定好的最适浓度加入。取50.0 μL已准备好的细胞悬液与50.0 μL混合抗体混匀，于冰上避光孵育30 min，加入1 mL PBS，400 g 5 min清洗，弃上清，加入300 μL PBS重悬，经流式细胞仪检测，记录约1×10^6^个Events，通过Flowjo软件进行数据分析。

### 1.5 统计学方法

本研究所有统计学分析及绘图均采用R 4.2.1进行。比较不同疗效组别间指标差异，分类计数资料采用例数（%）表示，采用卡方检验或Fisher确切检验分析；定量数据以均数±标准差（Mean±SD）或中位数（Q1, Q3）表示，正态分布数据采用t检验，非正态分布数据采用Wilcoxon秩和检验分析。通过绘制受试者工作特征（receiver operating characteristic, ROC）曲线确定外周血免疫细胞因子在预测晚期肺癌免疫治疗疗效中的cut-off值，并通过Logistic回归分析确定患者免疫治疗疗效的影响因素。绘制Kaplan-Meier曲线并利用Log-rank检验比较组间PFS差异。通过Cox风险比例模型进行单因素与多因素分析校正其他变量后PFS的预后影响因素。检验水准为P<0.05。

## 2 结果

### 2.1 患者临床病理特征

本临床研究共纳入69例晚期肺癌患者。其中男性61例（88.4%），女性8例（11.6%），年龄≥65岁25例（36.2%），<65岁44例（63.8%）。其中腺癌34例（49.3%），鳞癌20例（29.0%），其他病理类型15例（21.7%）。所有入组患者接受抗PD-1/PD-L1免疫治疗，66例（95.7%）患者接受化疗联合免疫治疗，3例（4.3%）患者接受免疫单药治疗。患者接受治疗后R组42例（60.9%），NR组27例（39.1%）。流式队列是来自总临床研究队列中的27例患者，其中男性24例（88.9%），女性3例（11.1%），年龄≥65岁10例（37.0%），年龄<65岁17例（63.0%），R组13例（48.1%），NR组14例（51.9%）。表1为本研究队列患者不同疗效组别临床病理特征，R组与NR组患者在年龄、性别、吸烟史等临床病理特征方面无显著差异（P>0.05），基线可比。

**表1:** 晚期肺癌患者临床病理特征及实验室检查结果

Variables	Clinical cohort				FACS cohort			
	Total (n=69)	NR (n=27)	R (n=42)	P	Total (n=27)	NR (n=14)	R (n=13)	P
Age, yr, n (%)				0.510				>0.999
≥65	25 (36.2)	8 (29.6)	17 (40.5)		10 (37.0)	5 (35.7)	5 (38.5)	
<65	44 (63.8)	19 (60.4)	25 (59.5)		17 (63.0)	9 (64.3)	8 (61.5)	
Gender, n (%)				0.467				0.596
Female	8 (11.6)	2 (7.4)	6 (14.3)		3 (11.1)	1 (7.1)	2 (15.4)	
Male	61 (88.4)	25 (92.6)	36 (85.7)		24 (88.9)	13 (92.9)	11 (84.6)	
Smoking history, n (%)				>0.999				0.596
No	14 (20.3)	5 (18.5)	9 (21.4)		4 (14.8)	3 (21.4)	1 (7.7)	
Yes	55 (79.7)	22 (81.5)	33 (78.6)		23 (85.2)	11 (78.6)	12 (92.3)	
Family history, n (%)				0.079				0.098
No	60 (87.0)	26 (96.3)	34 (81.0)		24 (88.9)	14 (100.0)	10 (76.9)	
Yes	9 (13.0)	1 (3.7)	8 (19.0)		3 (11.1)	0 (0.0)	3 (23.1)	
TNM stage, n (%)				0.885				>0.999
III	25 (36.2)	9 (33.3)	16 (38.1)		9 (33.3)	5 (35.7)	4 (30.8)	
IV	44 (63.8)	18 (66.7)	26 (61.9)		18 (66.7)	9 (64.3)	9 (69.2)	
Histology, n (%)				0.959				0.472
LUAD	34 (49.3)	13 (48.1)	21 (50.0)		19 (70.4)	11 (78.6)	8 (61.5)	
LUSC	20 (29.0)	8 (29.6)	12 (28.6)		6 (22.2)	3 (21.4)	3 (23.1)	
NSCLC	4 (5.8)	1 (3.7)	3 (7.1)		2 (7.4)	0 (0.0)	2 (15.4)	
SCLC	11 (15.9)	5 (18.5)	6 (14.3)					
Type of immunotherapy, n (%)				0.628				0.836
Atezolizumab	1 (1.4)	0 (0.0)	1 (2.4)		0 (0.0)	0 (0.0)	0 (0.0)	
Camrelizumab	12 (17.4)	6 (22.2)	6 (14.3)		8 (29.6)	4 (28.6)	4 (30.8)	
Cejemly	1 (1.4)	0 (0.0)	1 (2.4)		1 (3.7)	0 (0.0)	1 (7.7)	
Durvalumab	6 (8.7)	2 (7.4)	4 (9.5)		0 (0.0)	0 (0.0)	0 (0.0)	
Pembrolizumab	17 (24.6)	5 (18.5)	12 (28.6)		5 (18.5)	2 (14.3)	3 (23.1)	
Sintilimab	8 (11.6)	2 (7.4)	6 (14.3)		4 (14.8)	2 (14.3)	2 (15.4)	
SlurryMab	2 (2.9)	1 (3.7)	1 (2.4)		0 (0.0)	0 (0.0)	0 (0.0)	
Tislelizumab	20 (29.0)	9 (33.3)	11 (26.2)		9 (33.3)	6 (42.9)	3 (23.1)	
Toripalimab	2 (2.9)	2 (7.4)	0 (0.0)		0 (0.0)	0 (0.0)	0 (0.0)	
Type of chemotherapy, n (%)				0.813				0.236
AC	31 (44.9)	13 (48.1)	18 (42.9)		18 (66.7)	11 (78.6)	7 (53.8)	
EC	11 (15.9)	5 (18.5)	6 (14.3)		0 (0.0)	0 (0.0)	0 (0.0)	
TP	1 (1.4)	0 (0.0)	1 (2.4)		0 (0.0)	0 (0.0)	0 (0.0)	
T	1 (1.4)	0 (0.0)	1 (2.3)		0 (0.0)	0 (0.0)	0 (0.0)	
TC	22 (31.9)	7 (25.9)	15 (35.7)		9 (33.3)	3 (21.4)	6 (46.2)	
NO	3 (4.3)	2 (2.9)	1 (1.4)		0 (0.0)	0 (0.0)	0 (0.0)	
Six months RECIST, n (%)				<0.001				<0.001
PD	27 (39.1)	27 (100.0)	0 (0.0)		14 (51.9)	14 (100.0)	0 (0.0)	
PR	38 (55.1)	0 (0.0)	38 (90.5)		13 (48.1)	0 (0.0)	13 (100.0)	
SD	4 (5.8)	0 (0.0)	4 (9.5)		0 (0.0)	0 (0.0)	0 (0.0)	
	Total (n=69)	NR (n=27)	R (n=42)	P	Total (n=27)	NR (n=14)	R (n=13)	P
B2 WBC, n (%)				<0.001				0.046
≥8.275×10^9^/L	18 (26.1)	14 (51.9)	4 (9.5)		10 (37.0)	8 (57.1)	2 (15.4)	
<8.275×10^9^/L	51 (73.9)	13 (48.1)	38 (90.5)		17 (63.0)	6 (42.9)	11 (84.6)	
B2 LYMPH%, n (%)				0.006				0.033
≥31.550	19 (27.5)	2 (7.4)	17 (40.5)		7 (25.9)	1 (7.1)	6 (46.2)	
<31.550	50 (72.5)	25 (92.6)	25 (59.5)		20 (74.1)	13 (92.9)	7 (53.8)	
B2 CYFRA21-1, n (%)^a^				<0.001				0.070
≥4.255 ng/mL	20 (37.7)	12 (80.0)	8 (21.1)		8 (42.1)	6 (66.7)	2 (40.0)	
<4.255 ng/mL	33 (62.3)	3 (20.0)	30 (78.9)		11 (57.9)	3 (33.3)	8 (60.0)	
B2 NLR, n (%)				0.011				0.077
≥1.738	51 (73.9)	25 (92.6)	26 (61.9)		21 (77.8)	13 (92.9)	8 (61.5)	
<1.738	18 (26.1)	2 (7.4)	16 (38.1)		6 (22.2)	1 (7.1)	5 (38.5)	

^a^: The CYFRA21-1 level was not detected in all the patients and only the patients detected were enrolled in this analysis.

FACS: fluorescence activating cell sorter; R: response; NR: no response; PD: progressive disease; CR: complete response; PR: partial response; SD: stable disease; PFS: progression-free survival; LUAD: lung adenocarcinoma; LUSC: lung squamous cell carcinoma; NSCLC: non-small cell lung cancer; SCLC: small cell lung cancer; CT: chemotherapeutic; IO: immunotherapy; AC: pemetrexed plus carboplatinum; EC: etoposide plus carboplatinum; TC: paclitaxel plus carboplatinum; TP: paclitaxel plus cisplatinum; NO: vinorelbine plus oxaliplatin; TNM: tumor-node-metastasis; WBC: white blood cell; LYMPH%: lymphocyte percentage; CYFRA21-1: cytokeratin 19 fragment; NLR: neutrophil-to-lymphocyte ratio; RECTIST: Response Evaluation Criteria in Solid Tumors; B2: 1 cycle after treatment.

### 2.2 临床队列外周血实验室检测指标与免疫治疗疗效的相关性

共有42例（60.9%）患者在接受免疫治疗后获得持续6个月以上的临床缓解。收集患者治疗前基线（B1）与1个周期治疗后（B2）的实验室检查结果，对比R组和NR组治疗前及治疗1个周期后外周血检测指标的差异，结果显示治疗1个周期后R组患者的LYMPH%（P=0.006）高于治疗NR组。而治疗1个周期后有R组患者的肿瘤标志物CYFRA21-1（P<0.001）、WBC（P<0.001）、NLR（P=0.011）明显低于治疗NR组（表1）。采用ROC曲线，确定第1个周期治疗后外周血的WBC的cut-off值为8.275×10^9^/L（图1A），高于8.275×10^9^/L的患者为高水平组；CYFRA21-1的cut-off值为4.255 ng/mL（图1B），高于4.255 ng/mL的患者为高水平组；LYMPH%的cut-off值为31.550%（图1C），高于31.850%的患者为高水平组；NLR的cut-off值为1.738（图1D），高于1.738的患者为高水平组。将患者治疗1个周期后外周血的CYFRA21-1、LYMPH%、WBC、NLR以及年龄、吸烟史、肿瘤家族史等指标纳入Logistic单因素分析（表2），结果显示治疗1个周期后高CYFRA21-1（OR=15.00, 95%CI: 3.40-66.00, P<0.001）、高WBC（OR=13.00, 95%CI: 2.80-63.00, P=0.001）、高NLR（OR=10.00, 95%CI: 1.20-86.00, P=0.033）是免疫治疗疗效的危险因素，高LYMPH%（OR=0.09, 95%CI: 0.01-0.74, P=0.025）是免疫治疗疗效的保护因素（表2）。

**表2:** 晚期肺癌患者疗效与外周血指标单因素Logistic回归分析

Variables	Clinical cohort		FACS cohort	
	OR (95%CI)	P	OR (95%CI)	P
Age (<65 yr vs ≥65 yr)	0.77 (0.22-2.70)	0.680	0.89 (0.19-4.20)	0.880
Smoking history (Yes vs No)	0.27 (0.03-2.40)	0.230	3.30 (0.29-36.00)	0.330
Family history (Yes vs No)	5.8e+07 (0-Inf)	0.990	6e+07 (0-Inf)	0.990
Clincial stage (IV vs III)	0.92 (0.27-3.10)	0.890	1.20 (0.25-6.20)	0.790
B2 WBC (Low vs High)	13.00 (2.80-63.00)	0.001	7.30 (1.20-46.00)	0.034
B2 LYMPH% (Low vs High)	0.09 (0.01-0.74)	0.025	0.09 (0.01-0.90)	0.041
B2 CYFRA21-1 (Low vs High)	15.00 (3.40-66.00)	<0.001	8.00 (1.00-64.00)	0.050
B2 NLR (Low vs High)	10.00 (1.20-86.00)	0.033	8.10 (0.80-83.00)	0.077
B1 TCF1^+^CD8^+ ^T cells (Low vs High)			0.05 (0.00-0.49)	0.010
B2 PD-1^+^CD4^+ ^T cells (Low vs High)			0.12 (0.02-0.69)	0.017

B1: baseline; TCF1: transcription factor T cell factor 1; PD-1: programmed death receptor 1; OR: odds ratio.

**图1:**
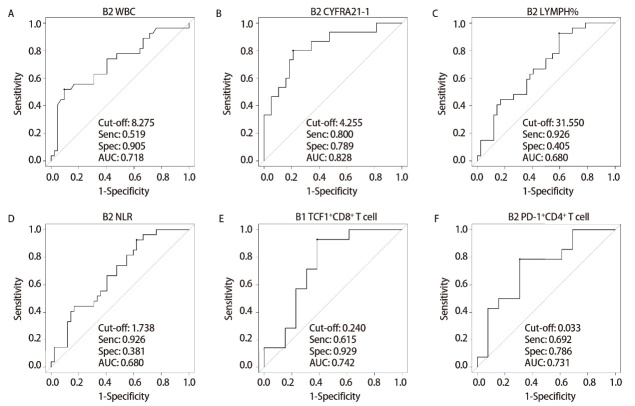
晚期肺癌患者外周血指标预测6个月临床缓解的ROC曲线。A：治疗1个周期后（B2），69例晚期肺癌患者WBC预测反应者的ROC曲线；B：治疗1个周期后（B2），53例晚期肺癌患者CYFRA21-1预测反应者的ROC曲线；C：治疗1个周期后（B2），69例晚期肺癌患者LYMPH%预测反应者的ROC曲线；D: 治疗1个周期后（B2），69例晚期肺癌患者NLR预测反应者的ROC曲线；E：流式队列27例患者外周血治疗前（B1）TCF1^+^CD8^+ ^T细胞频率预测反应者的ROC曲线；F：治疗1个周期后（B2），流式队列患者外周血PD-1^+^CD4^+ ^T细胞频率预测反应者的ROC曲线。

### 2.3 临床队列外周血实验室检测指标与患者PFS的相关性

69 例晚期肺癌患者中位PFS为250 d。对PFS进行单因素和多因素Cox回归分析，发现治疗1个周期后低水平的WBC可以降低进展风险（HR=0.44, 95%CI: 0.23-0.84, P=0.013），治疗后低水平的CYFRA21-1（HR=0.51, 95%CI: 0.26-1.00, P=0.049）的患者有更长的PFS，详见表3。根据Cox回归发现的影响预后的因素分组绘制相应的生存曲线图（图2A-图2C）。发现治疗1个周期后WBC<8.275×10^9^/L组的患者相较于WBC≥8.275×10^9^/L组有更长的PFS（P<0.001），治疗1个周期后LYMPH%≥31.550%的患者PFS显著长于低水平组（P=0.041），NLR<1.738的患者比高水平组的患者有更长的PFS（P=0.030）。

**表3:** 晚期肺癌患者PFS相关单因素和多因素分析

Variables	Univariate analysis		Multivariate analysis	
	HR (95%CI)	P	HR (95%CI)	P
Age (<65 yr vs ≥65 yr)	1.58 (0.86-2.89)	0.138		
Smoking history (Yes vs No)	1.02 (0.51-2.05)	0.948		
Family history (Yes vs No)	0.51 (0.20-1.28)	0.150		
Pathological subtype				
LUSC (LUSC vs LUAD)	1.16 (0.60-2.23)	0.659		
NSCLC (NSCLC vs LUAD)	1.13 (0.34-3.74)	0.854		
SCLC (SCLC vs LUAD)	1.72 (0.82-3.63)	0.154		
Clinical stage (IV vs III)	0.94 (0.53-1.67)	0.843		
B2 WBC (Low vs High)	0.33 (0.18-0.61)	<0.001	0.44 (0.23-0.84)	0.013
B2 LYMPH% (Low vs High)	1.99 (1.02-3.88)	0.045	0.37 (0.05-2.89)	0.345
B2 CYFRA21-1 (Low vs High)	0.51 (0.27-0.99)	0.048	0.51 (0.26-1.00)	0.049
B2 NLR (Low vs High)	0.47 (0.24-0.94)	0.033	0.25 (0.03-2.04)	0.196

HR: hazard ratio.

**图2:**
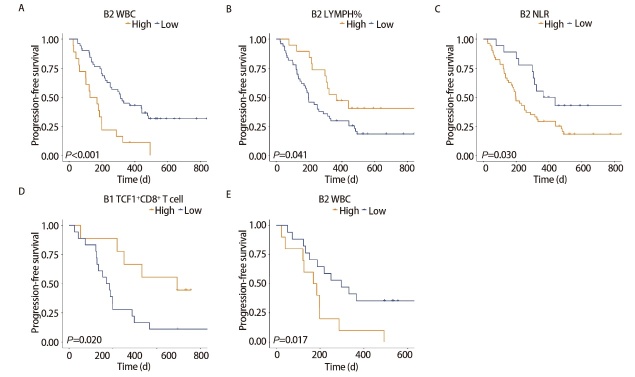
晚期肺癌患者PFS的Kaplan-Meier生存分析。A：用药1个周期后（B2），按WBC分组的患者PFS的Kaplan-Meier生存曲线；B：用药1个周期后（B2），按LYMPH%分组的患者PFS的Kaplan-Meier生存曲线；C：用药1个周期后（B2），按NLR分组的患者PFS的Kaplan-Meier生存曲线；D：治疗前（B1），按外周血TCF1^+^CD8^+^ T 细胞分组的患者PFS的Kaplan-Meier生存曲线；E：用药1个周期后（B2），按WBC细胞分组的患者PFS的Kaplan-Meier生存曲线。

### 2.4 外周血淋巴细胞亚群与疗效的相关性

对本研究队列中27例具有治疗前后配对的外周血样本进行多色流式细胞术，分析淋巴细胞亚群与治疗反应的相关性。对比分析R组和NR组治疗前及治疗1个周期后外周血检测指标的差异，结果显示治疗前R组患者的基线TCF1^+^CD8^+ ^T淋巴细胞占比（P=0.035）高于治疗NR组。治疗1个周期后PD-1^+^CD4^+ ^T淋巴细胞在R组患者中显著更高（P=0.043）（表4），而相应指标在治疗前基线样本中未发现显著差异。采用ROC曲线，确定基线（B1）外周血的TCF1^+^CD8^+ ^T淋巴细胞百分比的cut-off值为24%（图1E），高于24%的患者为高水平组；治疗1个周期后（B2）PD-1^+^CD4^+ ^T淋巴细胞百分比的cut-off值为3.3%（图1F），高于3.3%的患者为高水平组。将基线TCF1^+^CD8^+ ^T淋巴细胞占比、治疗1个周期后PD-1^+^CD4^+ ^T淋巴细胞占比、CYFRA21-1、LYMPH%以及WBC等指标作为变量纳入Logistic回归模型，结果显示，治疗1个周期后高WBC（OR=7.30, 95%CI: 1.20-46.00, P=0.034）是免疫治疗反应的危险因素，基线外周血中高水平TCF1^+^CD8^+ ^T淋巴细胞占比（OR=0.05, 95%CI: 0.0047-0.49, P=0.010）、治疗1个周期后高LYMPH%（OR=0.09, 95%CI: 0.01-0.90, P=0.041）和高水平PD-1^+^CD4^+ ^T淋巴细胞占比（OR=0.12, 95%CI: 0.02-0.69, P=0.017）是免疫治疗反应的保护性因素（表2）。

**表4:** 流式队列患者疗效与外周血指标的相关性分析

Variables	Total (n=27)	NR (n=14)	R (n=13)	P
B1 CD19^+ ^B cells, Mean±SD	0.07±0.04	0.07±0.04	0.08±0.05	0.969
B1 CD3^+ ^T cells, Mean±SD	0.50±0.16	0.49±0.14	0.51±0.18	0.750
B1 CD8^+ ^T cells, Mean±SD	0.40±0.14	0.44±0.14	0.35±0.13	0.085
B1 CD4^+ ^T cells, Mean±SD	0.46±0.12	0.44±0.12	0.49±0.12	0.222
B2 CD19^+ ^B cells, Median (Q1, Q3)	0.06 (0.04, 0.09)	0.07 (0.05, 0.08)	0.05 (0.04, 0.10)	0.905
B2 CD3^+ ^T cells, Mean±SD	0.53±0.20	0.49±0.19	0.57±0.20	0.252
B2 CD8^+ ^T cells, Mean±SD	0.42±0.13	0.44±0.14	0.40±0.12	0.416
B2 CD4^+ ^T cells, Mean±SD	0.46±0.11	0.45±0.14	0.46±0.08	0.766
B1 TCF1^+^CD8^+ ^T cells, Median (Q1, Q3)	0.14 (0.10, 0.26)	0.12 (0.08, 0.20)	0.26 (0.12, 0.41)	0.035
B1 PD-1^+^CD4^+ ^T cells, Median (Q1, Q3)	0.1 (0.08, 0.16)	0.09 (0.07, 0.14)	0.1 (0.09, 0.16)	0.544
B2 TCF1^+^CD8^+ ^T cells, Median (Q1, Q3)	0.15 (0.11, 0.20)	0.12 (0.09, 0.19)	0.19 (0.14, 0.29)	0.132
B2 PD-1^+^CD4^+ ^T cells, Median (Q1, Q3)	0.03 (0.01, 0.04)	0.01 (0.01, 0.03)	0.04 (0.02, 0.06)	0.043

### 2.5 外周血淋巴细胞亚群与患者PFS的相关性

流式队列27例晚期肺癌患者中位PFS为199 d。对PFS进行单因素和多因素Cox回归分析，发现基线低水平TCF1^+^CD8^+ ^T淋巴细胞占比的疾病进展风险更高（HR=4.84, 95%CI: 1.44-16.31, P=0.011），治疗1个周期后低水平WBC（HR=0.32, 95%CI: 0.11-0.93, P=0.037）和低水平CYFRA21-1（HR=0.16, 95%CI: 0.05-0.54, P=0.003）可以降低患者疾病进展风险，详见表5。根据Cox回归发现的影响预后的因素分组绘制相应的生存曲线图（图2D-图2E）。发现基线TCF1^+^CD8^+ ^T淋巴细胞占比>24%的患者的PFS显著长于低水平组患者（P=0.020），治疗1个周期后WBC<8.275×10^9^/L组的患者相较于WBC≥8.275×10^9^/L组有更长的PFS（P=0.017）。

**表5:** 流式队列晚期肺癌患者PFS相关单因素和多因素分析

Variables	Univariate analysis		Multivariate analysis	
	HR (95%CI)	P	HR (95%CI)	P
Age (<65 yr vs ≥65 yr)	1.15 (0.46-2.85)	0.770		
Smoking history (Yes vs No)	0.57 (0.19-1.72)	0.317		
Family history (Yes vs No)	0.19 (0.03-1.46)	0.111		
Pathological subtype				
LUSC (LUSC vs LUAD)	0.72 (0.24-12.18)	0.565		
NSCLC (NSCLC vs LUAD)	0.36 (0.05-2.70)	0.318		
Clinical stage (IV vs III)	1.05 (0.28-4.02)	0.938		
B2 WBC (Low vs High)	0.35 (0.15-0.86)	0.021	0.32 (0.11-0.93)	0.037
B2 LYMPH% (Low vs High)	2.52 (0.84-7.56)	0.099		
B2 CYFRA21-1 (Low vs High)	0.33 (0.12-0.94)	0.037	0.16 (0.05-0.54)	0.003
B2 NLR (Low vs High)	0.33 (0.09-1.13)	0.076		
B1 TCF1^+^CD8^+ ^T cells (Low vs High)	3.19 (1.14-8.90)	0.027	4.84 (1.44-16.31)	0.011
B2 PD1^+^CD4^+ ^T cells (Low vs High)	0.74 (0.73-4.17)	0.214		

## 3 讨论

大部分肺癌患者确诊时已处于病情中晚期阶段，丧失手术根治性切除病灶的机会。以抗PD-1/PD-L1为主的免疫检查点抑制剂联合化疗治疗已经成为驱动基因阴性中晚期肺癌的一线标准治疗，在大量的临床研究中都显示出优于化疗的效果^[14⇓⇓-17]^，能够有效延长肺癌患者总生存期及PFS。然而，只有一少部分患者可从免疫治疗中获益，而目前尚缺乏用于指导临床实践的生物标志物。目前对于免疫治疗生物标志物的探索，大多研究者都关注于在肿瘤微环境、肿瘤免疫和循环肿瘤DNA等层面的研究，但肿瘤组织取材具有较大难度，无法作为常规临床监测方案。近期有不少研究关注到免疫治疗过程中外周血指标的动态变化，提示外周血免疫细胞状态与免疫治疗反应的相关性^[12,13,18]^，因此本研究收集了入组中晚期肺癌患者的临床病理学资料，结合临床常规的血液学标志物结果，探索出一些与PD-1/PD-L1抑制剂疗效及预后相关的因素。以期望能够筛选出中晚期肺癌患者中最可能从免疫治疗中获益的人群，为临床用药决策提供一些思路。

本研究的疗效相关性分析结果提示相对于NR组的患者而言，治疗1个周期后R组患者有显著更高的LYMPH%，而治疗1个周期后肿瘤标记物CYFRA21-1、WBC及NLR水平明显更低。推测可能与免疫治疗有效地激活了机体的免疫系统并缩减了患者肿瘤负荷有关。此外，治疗早期的外周血标志物能够很好地预测患者6个月的临床获益，这与既往文献^[8,19,20]^报道的早期外周血指标具有预测免疫治疗疗效的价值的观点一致。在预后分析结果中，治疗1个周期后LYMPH%、WBC、CYFRA21-1、NLR可以预测PFS，且WBC与CYFRA21-1是PFS的独立预测因子。

总研究队列中发现在治疗1个周期后外周血LYMPH%与治疗反应显著相关，本研究进一步探索与免疫疗效及预后相关的外周血细胞亚群。前瞻性地采集了27例患者新鲜外周血样本进行流式细胞术的检测。通过分析患者治疗前后的纵向外周血发现基线TCF1^+^CD8^+^ T细胞与治疗1个周期后PD-1^+^CD4^+^ T细胞与免疫治疗反应显著相关，并且基线TCF1^+^CD8^+^ T细胞占比是中晚期肺癌患者PFS的独立影响因素。我们的结果提示，基线TCF1^+^CD8^+^ T细胞占比更高的患者对免疫检查点抑制剂治疗有更好的反应并且PFS更长。既往小鼠研究也提到TCF1^+^干性记忆T细胞亚群可能是肿瘤免疫微环境中T细胞的主要来源，可以自我更新以及分化，介导对免疫治疗的持续反应^[21⇓⇓-24]^。推测具有此类特征的患者更可能从免疫治疗中获益，但尚需研究揭示其反应机制。

本研究作为单中心前瞻性队列研究，符合标准的入组患者数量少，导致一定的集合偏倚，患者使用的免疫治疗药物和治疗周期不同，随访时间不同。另外，本研究入组患者中23%患者的CYFRA21-1水平结果未完善导致未能收集。因此需要更大规模多中心前瞻性研究来进一步验证本研究结论。

综上所述，本研究显示基线高TCF1^+^CD8^+^ T细胞占比、治疗1个周期后外周血LYMPH%更高、WBC、NLR及CYFRA21-1水平明显更低的中晚期肺癌患者更可能从免疫检查点抑制剂治疗中获益。未来可以通过更大样本量的研究进一步探索TCF1^+^CD8^+^ T细胞相关指标在晚期肺癌患者中的应用价值，为临床医生提供更方便有效的参考依据。

**Competing interests**

The authors declare that they have no competing interests.
